# Preoperative inspiratory muscle training to prevent postoperative pulmonary complications in patients undergoing esophageal resection (PREPARE study): study protocol for a randomized controlled trial

**DOI:** 10.1186/1745-6215-15-144

**Published:** 2014-04-27

**Authors:** Karin Valkenet, Jaap CA Trappenburg, Rik Gosselink, Meindert N Sosef, Jerome Willms, Camiel Rosman, Heleen Pieters, Joris JG Scheepers, Saskia C de Heus, John V Reynolds, Emer Guinan, Jelle P Ruurda, Els HE Rodrigo, Philippe Nafteux, Marianne Fontaine, Ewout A Kouwenhoven, Margot Kerkemeyer, Donald L van der Peet, Sylvia W Hania, Richard van Hillegersberg, Frank JG Backx

**Affiliations:** 1 Department of Rehabilitation, Nursing Science and Sports, University Medical Center Utrecht, PO Box 85500, Utrecht 3508 GA, The Netherlands; 2 Department of Rehabilitation Sciences, KU Leuven, University Hospital Leuven, Tervuursevest 101, Leuven 3001, Belgium; 3 Department of Surgery, Atrium Medical Center, PO Box 4446, Heerlen 6401 CX, The Netherlands; 4 Department of Physical Therapy, Atrium Medical Center, PO Box 4446, Heerlen 6401 CX, The Netherlands; 5 Department of Surgery, Canisius Wilhelmina Hospital, PO Box 9015, Nijmegen 6500 GS, The Netherlands; 6 Department of Physical Therapy, Canisius Wilhelmina Hospital, PO Box 9015, Nijmegen 6500 GS, The Netherlands; 7 Department of Surgery, Reinier de Graaf Hospital, PO Box 5011, Delft 2600 GA, The Netherlands; 8 Department of Physical Therapy, Reinier de Graaf Hospital, PO Box 5011, Delft 2600 GA, The Netherlands; 9 Department of Surgery, St James’s Hospital, Trinity Centre for Health Sciences, Dublin 8, Ireland; 10 Discipline of Physiotherapy, St James’s Hospital, Trinity Centre for Health Sciences, Dublin 8, Ireland; 11 Department of Surgery, University Medical Center Utrecht, PO Box 85500, Utrecht 3508 GA, The Netherlands; 12 Department of Thoracic Surgery, University Hospitals Leuven, Herestraat 49 3000, Leuven, Belgium; 13 Department of Physical Therapy, University Hospitals Leuven, Herestraat 49 3000, Leuven, Belgium; 14 Department of Surgery, Hospital Group Twente (ZGT), PO Box 7600, Almelo 7600 SZ, The Netherlands; 15 Department of Physical Therapy, Hospital Group Twente (ZGT), PO Box 7600, Almelo 7600 SZ, The Netherlands; 16 Department of Surgery, VU University Medical Center, PO Box 7057, Amsterdam 1007 MB, The Netherlands; 17 Department of Physical Therapy, VU University Medical Center, PO Box 7057, Amsterdam 1007 MB, The Netherlands

**Keywords:** Inspiratory muscle training, Physical therapy, Preoperative, Esophageal resection, Pneumonia

## Abstract

**Background:**

Esophageal resection is associated with a high incidence of postoperative pneumonia. Respiratory complications account for almost half of the readmissions to the critical care unit. Postoperative complications can result in prolonged hospital stay and consequently increase healthcare costs. In cardiac surgery a preoperative inspiratory muscle training program has shown to prevent postoperative pneumonia and reduce length of hospital stay. While in some surgical centers inspiratory muscle training is already used in the preoperative phase in patients undergoing esophageal resection, the added value of this intervention on the reduction of pulmonary complications has not yet been investigated in large surgical populations other than cardiac surgery in a randomized and controlled study design.

**Methods/Design:**

The effect of a preoperative inspiratory muscle training program on the incidence of postoperative pneumonia in patients undergoing esophageal resection will be studied in a single blind multicenter randomized controlled trial (the PREPARE study). In total 248 patients (age >18 years) undergoing esophageal resection for esophageal cancer will be included in this study. They are randomized to either usual care or usual care with an additional inspiratory muscle training intervention according to a high-intensity protocol which is performed with a tapered flow resistive inspiratory loading device. Patients have to complete 30 dynamic inspiratory efforts twice daily for 7 days a week until surgery with a minimum of 2 weeks. The starting training load will be aimed to be 60% of maximal inspiratory pressure and will be increased based on the rate of perceived exertion.

The main study endpoint is the incidence of postoperative pneumonia. Secondary objectives are to evaluate the effect of preoperative inspiratory muscle training on length of hospital stay, duration of mechanical ventilation, incidence of other postoperative (pulmonary) complications, quality of life, and on postoperative respiratory muscle function and lung function.

**Discussion:**

The PREPARE study is the first multicenter randomized controlled trial to evaluate the hypothesis that preoperative inspiratory muscle training leads to decreased pulmonary complications in patients undergoing esophageal resection.

**Trial registration:**

NCT01893008.

## Background

The numbers of esophageal cancer patients are increasing worldwide. The incidence of esophageal cancer is increased by 50% in the past two decades [1]. Surgical resection, mostly preceded by neoadjuvant chemoradiotherapy, is regarded the only curable option for esophageal cancer [2]. While the in-hospital mortality rate is below 5% after this surgery, several studies report in-hospital postoperative pneumonia rates around 30% [2-4]. Respiratory complications account for almost half of the readmissions to critical care units [5] and the development of pneumonia is associated with a mortality of 20% compared with 3% among patients free of pneumonia [6]. Besides increased mortality, the high rate of pneumonia results in increased length of stay in the intensive care unit (ICU) and in the hospital [7,8] which results in increased and prolonged use of medical care, with subsequent consequences for healthcare costs. Therefore, preventing pneumonia is better than curing pneumonia.

Several factors associated with esophageal resection, such as diaphragmatic dysfunction as a result of intra-thoracic manipulation of tissue, perioperative lung collapse, mechanical ventilation, and supine positioning, can increase perioperative ventilatory demand [9]. When the ventilatory demand (or workload) exceeds the ventilatory capacity (or ability), this imbalance can lead to ventilatory pump failure. Following, ventilatory pump failure can result in alveolar hypoventilation which may progress to respiratory failure, pneumonia, or even death [9].

Physical therapy interventions to prevent pneumonia and other postoperative pulmonary complications (PPCs) often focus on postoperative prophylaxis using various respiratory techniques and devices [10-12]. A relatively new physical therapy intervention to prevent PPCs and pneumonia is preoperative inspiratory muscle training (IMT). IMT is performed with an inspiratory loading device and results in increased strength and endurance of the inspiratory muscles [13-15]. Since the ventilatory capacity partly depends on optimal respiratory muscle performance, increased inspiratory muscle function may lead to improved ventilatory capacity [9]. The hypothesis is that when IMT is applied in the preoperative phase of esophageal resection it may prevent the imbalance between ventilatory demand and ventilatory capacity resulting in a reduction of PPCs.

A large randomized controlled trial (RCT) found that IMT before coronary bypass surgery significantly reduced PPCs and length of hospital stay. PPCs, including pneumonia, were reduced by over 50% in patients at high risk of developing pulmonary complications [13]. In abdominal surgery beneficiary effects are reported on the incidence of atelectasis [16].

While in some surgical centers IMT is already used in the preoperative phase in patients undergoing esophageal resection, the effect of this intervention has not yet been investigated in a randomized controlled study in large surgical populations other than cardiac surgery. One non-randomized pilot study concluded that IMT is feasible and well tolerated before esophageal resection but did not show an effect on postoperative pulmonary outcome or length of hospital stay [15].

This article provides a detailed description of the background, the targeted population, and study methodology of the PREPARE study (preoperative inspiratory muscle training to prevent postoperative pneumonia in patients undergoing esophageal resection), a multicenter RCT investigating the effect of preoperative IMT in esophageal resection candidates.

## Methods/Design

### Study objectives

The primary objective is to evaluate the effect of preoperative IMT in addition to usual care, compared to usual care without IMT, in patients undergoing esophageal resection on the incidence of postoperative pneumonia. Secondary objectives are to evaluate the effect of preoperative inspiratory muscle training on length of hospital stay, duration of mechanical ventilation, incidence of other postoperative (pulmonary) complications, quality of life, and on postoperative respiratory muscle function and lung function.

### Study design and setting

This study is conducted as a single-blind multicenter RCT and will be coordinated by the University Medical Center Utrecht (UMCU) (Department of Rehabilitation, Nursing Science & Sports and Department of Surgery). Patients are randomly assigned to either care as usual without IMT or to IMT as an addition to care as usual. The randomization procedure is performed by a flexible web-based randomization system. The assignment sequence will be concealed. Randomization is stratified by center and minimization techniques are applied for surgical technique (transhiatal esophageal resection, transthoracic esophageal resection, minimally invasive esophageal resection).

This study is funded by ‘NutsOhra Foundation’ and ‘The Friends of the UMC Utrecht Foundation’ , and will be conducted in accordance with the principles of the Declaration of Helsinki and Good Clinical Practice Guidelines. The independent ethics committee of the UMCU has approved the study. Written informed consent will be obtained from all participating patients.

### Study population

All patients diagnosed with esophageal cancer and scheduled for esophageal resection with gastric conduit reconstruction by either a transhiatal esophageal resection (THE), a transthoracic esophageal resection (TTE), or minimally invasive (robot-assisted or conventional) thoraco-laparoscopic esophageal resection (MIE) are considered eligible for inclusion.

Further inclusion criteria are: (1) the patient is (cognitively) capable to understand and perform a preoperative IMT program; (2) the operation is scheduled at least 2 weeks after signing informed consent, since the patients need to be able to follow the intervention program for at least 2 weeks; and (3) the patient is willing to sign the informed consent form.

Patient are excluded based on the following criteria: (1) the patient is unable to communicate in the Dutch language; (2) age <18 years; and (3) the patient is participating in a conflicting trial concerning esophageal resection.

### Recruitment and informed consent

Patients are recruited from eight esophageal resection hospitals (in The Netherlands: Atrium Medical Center Heerlen, Canisius Wilhelmina Hospital Nijmegen, Reinier de Graaf Group Delft, University Medical Center Utrecht, VU Medical Center Amsterdam, Hospital Group Twente Almelo; in Belgium: University Hospital Gasthuisberg Leuven; and in Ireland: St. James’s Hospital Dublin). In every participating center one oncological surgeon and one physical therapist (PT) are the contact people for the coordinating researcher. Inclusion takes place for a period of approximately 1 year (September 2013 to August 2014). The local oncological surgeon determines whether a patient is eligible for inclusion in the PREPARE trial based on the inclusion criteria. At the start of the chemoradiation period or at the time the patient is informed about the planned surgery (when chemoradiation is not indicated), the surgeon informs the patient about the trial and hands over the trial information letter. After at least 5 days the patient is contacted by phone. When the patient is motivated to participate, an appointment with the local assessor is scheduled (after the chemoradiation period) for signing the informed consent form and completing the baseline measurements. The baseline measurement is followed by randomization, and the IMT instruction for patients in the intervention group. Both performed by the involved PT.

### Baseline assessment and follow-up measurements

The patient characteristics age, weight, height, gender, daily work, highest education, civil status, and preoperative physical therapy guidance (outside trial context) are prospectively recorded by means of a standardized interview during the baseline assessment. Data obtained from the patients’ medical records include history of COPD, smoking, pneumonia, diabetes mellitus, productive cough and cardiac events, tumor location, neoadjuvant treatment, prophylactic antibiotics, American Society of Anesthesiologists (ASA) score, admission and discharges dates, operation technique.

To evaluate the effect of IMT on respiratory muscle function and lung function, participants undergo measurements at five time points (baseline (T0), the day before surgery (T1), and during hospital stay on postoperative day 3 (T2), day 6 (T3), and day 9 (T4)). To determine an effect of IMT on quality of life (QoL) the EuroQol-5D and the SF-12 are assessed at baseline and 4 weeks after surgery. See for detailed description the paragraph secondary endpoints.

### Usual care

Surgical usual care in all Dutch centers consists of 5 to 6 weeks of preoperative chemoradiotherapy according to the CROSS group protocol [2]. In the centers in Belgium and Ireland, a different chemoradiotherapy schedule is used which is indicated on the tumor stage. The chemoradiotherapy schedule is followed by a recovery period of 4 to 8 weeks, depending on the recovery of the patient and waiting time for surgery per center. Patients not receiving chemoradiotherapy, will be placed on a waiting list for surgery which is in general at least 4 weeks. Physical therapy usual care in the preoperative phase can consist of ‘a preoperative instruction about postoperative physical therapy involvement’ , ‘preoperative advice to stay active or seek training guidance’ , ‘preoperative exercise guidance’ , or ‘no involvement until surgery’. Centers where preoperative IMT is currently part of usual care for all or a selected group of patients adapt to the PREPARE protocol and release IMT from their usual care. Patients assigned to the control group will only receive usual care.

Postoperatively physical therapy involvement can consist of respiratory physical therapy and physical therapy to facilitate early mobilization and independent functioning in activities of daily living. The differences in usual care per center is carefully registered.

### Intervention

Patients included in the intervention group receive an IMT program as an addition to usual care, starting in the recovery period after chemoradiotherapy. Patients not receiving chemoradiotherapy, start with the IMT program during the waiting period before surgery. IMT instructions are given by a specially instructed PT. The intervention used in this study entails the use of a tapered flow resistive inspiratory loading device: the POWERbreathe K3-series, an electronic IMT, and monitoring system [17]. It allows storing the data of the unsupervised training sessions to control compliance with the treatment.

The IMT intervention is based on the protocol of Bailey et al. [18]. This protocol is tailored individually. Starting inspiratory load is aimed at 60% of the measured maximal inspiratory pressure (Pi_max_). The load is incrementally increased based on the rate of perceived exertion (RPE) scored on a scale from 0 to 10 which is scored by the patient after each training session [19,20]. When patients score an RPE below 7, the inspiratory load of the threshold device will be increased with 5% by the patient. Patients have to complete 30 dynamic inspiratory efforts twice daily.

All patients in the intervention group are instructed during a face-to-face instruction session, will train at home 7 days a week for at least 2 weeks and continue training until surgery. Additionally, patients receive an instruction movie to watch at home and a training diary where the use of the inspiratory muscle trainer is illustrated step by step. Training results (RPE, level, load, power, volume, t-index) are recorded by the patients in the training diary after each training session. After the instruction session, the PT contacts the patient by phone after 3 days. When the training is not performed as instructed, the PT makes a follow-up appointment with the patient to repeat the instruction. Subsequently, training progress is evaluated during a weekly telephone consultation by the PT until surgery. At the end of the training period, IMT will be evaluated with a questionnaire. All involved PTs guiding patients during the IMT period received education and training during a meeting on the theory and practice of IMT organized by the research group.

### Blinding

The PREPARE study is a single blind randomized controlled trial. The local assessors (performing the informed consent procedure and the baseline and follow-up measurements), the surgeons, and other medical staff are blinded for group allocation. The patient and the PT (performing the randomization and guiding the intervention group) are not blinded for group allocation.

Patients in both groups will be informed that the PREPARE study aims to investigate the effect of a preoperative (home-based) IMT program on the reduction of postoperative pneumonia. The intervention group additionally receives detailed information about the intervention like timing, frequency, and intensity of the training. Included patients will not have contact with each other within the context of this trial.

### Primary endpoint

The primary outcome of the study is the incidence of postoperative pneumonia, as this is the most frequently occurring complication after esophageal resection and is an important cause of increased morbidity and mortality [6,21]. Pneumonia is defined according to the definition of the Utrecht Pneumonia Scoring System (UPSS) [22]. The UPSS corresponds with the clinical practice procedures for diagnosing pneumonia. Temperature, leukocyte count, and chest radiography are determinants that can objectively be assessed to diagnose pneumonia.

A score of 2 points or more (with at least 1 point assigned based on radiography) indicates treatment for pneumonia [22]. The UPSS variables will be scored in the participating centers on each day a pulmonary radiography has been performed. At the end of the trial, all chest radiographs will be reassessed by one blinded and independent radiologist. These scores will be used to determine the diagnosis of pneumonia in the context of this trial (Table 1).

**Table 1:** **The Utrecht Pneumonia scoring system**[22]

**Diagnostic determinant**	**Values**	**Score**
Temperature (°C)	≥ 36.1 and ≤ 38.4	0
	≥ 38.5 and ≤ 38.9	1
	≥ 39.0 and ≤ 36.0	2
Leukocyte count [×10^9^/L]	≥ 4.0 and ≤ 11.0	0
	< 4.0 or > 11.0	1
Pulmonary radiography	No infiltrate	0
	Diffused (or patchy) infiltrate	1
	Well-circumscribed infiltrate	2

### Secondary endpoints

**Figure 1:**
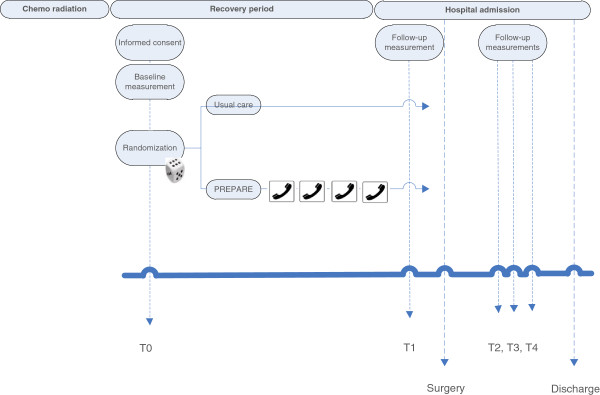
Flowchart of PREPARE procedures.

To evaluate the effect of IMT on respiratory muscle function and lung function, participants will undergo measurements at five time points (baseline (T0), the day before surgery (T1), and during hospital stay on postoperative day 3 (T2), day 6 (T3), and day 9 (T4) (Figure 1)). Forced expiratory volume in 1 second (FEV_1_) and forced vital capacity (FVC) are assessed using a portable spirometer. Respiratory muscle function will be evaluated using maximal inspiratory pressure (Pi_max_) and inspiratory muscle endurance (Pi_end_). Pi_max_ is measured at the mouth with a forceful inspiratory maneuver at residual volume. To measure Pi_end_ patients will be asked to breathe against an inspiratory load of 70% Pi_max_ until task failure (with a minimum of 3 minutes and a maximum of 7 minutes at baseline). All measurements are performed in the seated position.

To determine an effect of IMT on quality of life (QoL) the EuroQol-5D and the SF-12 are assessed at baseline and 4 weeks after surgery. Postoperative pulmonary complications other than pneumonia are scored postoperatively (for example, positive sputum cultures) as well as other postoperative complications (anastomotic leakage, chylus leakage, wound infection, cardiac complications, sepsis, vocal cord paralysis, critical illness polyneuropathy).

Other secondary endpoints are in-hospital mortality, length of postoperative hospital stay (starting on the day of surgery), mechanical ventilation time (number of hours spent on the mechanical ventilator during and directly following the primary surgery), and number of re-intubations.

### Other outcome measures

The following variables will be obtained from the patients’ medical records: discharge location (home, rehabilitation center, nursing home, other hospital, hospice), operation details (duration, location, technique, anastomosis, single/double lung ventilation, use of mini-tracheal cannula, blood loss, re-operation), mechanical ventilation details (duration, re-intubation), tumor type, Mandard score (tumor regression grade), classification of malignant tumors (TNM score), and date of first postoperative mobilization (active, standing aid, passive lift).

### Education and monitoring

All involved healthcare providers are well informed about the study procedures. Surgeons and specialized nurses providing the initial information about the PREPARE trial are personally informed about the aims and procedures of the study and their tasks within the trial by the coordinating researcher. The research group organized an afternoon meeting where the local assessors and PTs attended practical workshops about the procedures and use of all equipment. Additional written information is provided as well. Study procedures will be monitored by an independent monitor in the coordinating center and by the coordinating researcher in the participating centers during multiple monitoring visits (at the start of the trial, after the first three completed patients, after 1 year, and at the end of the trial).

### Sample size calculation

Sample size is calculated based on an expected reduction of 50% on the incidence of postoperative pneumonia [13]. Assuming a 30% incidence of postoperative pneumonia after esophageal resection in the control arm and using a significance level of 0.05 and a power of 80%, 118 patients are required in each arm [2,15,23]. Taking into account a 5% in-hospital mortality rate after surgery, approximately 124 patients per arm need to be included (248 patients in total).

### Statistical analyses

Data analyses will be performed in accordance with the intention to treat principle. IBM SPSS statistics software (version 20 or higher) will be used for data analyses. A two-sided *P* value of less than 0.05 is regarded to be statistically significant. Analyses will be adjusted for center.

Literature shows that the odds ratio (OR) can be interpreted as relative risk (RR) when the incidence of the outcome is < 10%. When the incidence is high, the OR can overestimate the RR [24-26]. Therefore, analyses of dichotomous variables will be performed using logistic regression analysis when the incidence is ≤ 10% and with Log-binomial regression analysis in the case of an incidence of > 10%. The incidence of pneumonia will be compared between the two groups and results will be presented as OR or RR with a 95% confidence interval (CI). The risk difference and numbers needed to treat will also be calculated when significant differences are found between groups.

For the continuous secondary outcome measures duration of mechanical ventilation and length of stay (ICU and total), survival analysis (Cox proportional hazards regression model) will be used to determine differences in time to extubation and discharge. For the categorical secondary outcome measures other pulmonary complications and in-hospital mortality the OR or RR will be calculated. Other outcomes (for example, lung function, respiratory muscle function, quality of life) will be compared between groups using linear regression for parametric data or the Mann–Whitney U Test for non-parametric data. To determine differences between time points in lung function and respiratory muscle function, results will be analyzed using the repeated measurements method (General Linear Modeling Repeated Measures). In case of missing measurements or covariates, multiple imputations will be performed.

## Discussion

The high incidence of pneumonia has large impact on postoperative outcomes after esophageal resection. Mortality rates are significantly higher compared to patients without pneumonia (20% *versus* 3%) [6] and the use of healthcare is substantially increased by extended duration of hospital stay associated with increased healthcare costs [5,7,8]. Furthermore, postoperative complications have a negative influence on long-term quality of life [27,28]. Less invasive surgical techniques attribute significantly to decreasing this high impact complication which is of high clinical relevance [4]. Preoperative IMT resulted in a reduction of 50% of postoperative pneumonia in cardiac surgery patients and might be a promising intervention to further reduce the incidence of postoperative pneumonia after esophageal resection [13]. The PREPARE study is the first multicenter RCT investigating the effect of this intervention in esophageal resection candidates.

Methodological decisions of the PREPARE trial are compared with strengths and weaknesses of other studies (since 2000) investigating the effect of preoperative IMT (Table 2).

**Table 2:** Overview of preoperative inspiratory muscle training studies

	**PREPARE trial**	**Dettling **[15]	**Agrelli **[29]	**Hulzebos **[13]	**Savci **[30]	**Dronkers **[16]	**Barbalho-Moulim **[31]	**Kulkarni **[32]
**Study population**	Esophageal resection	Esophageal resection	Esophageal resection	Cardiac surgery	Cardiac surgery	Upper abdominal surgery	Open bariatric surgery	Major abdominal surgery
**Number of (anticipated) included patients**	(248)	83	20	279	43	20	22	80
**Sample size calculation**	+	-	-	-	-	-	-	-
**Randomization**	Individual	-	-	Individual	Individual	Individual	Individual	Individual
**Control group without IMT**	+	+	-	+	+	+	+	+
**Pilot-study**	-	+	+	-	-	+	-	+
**Blinding**	Primary and secondary outcomes	-	-	Primary outcome	-	Primary outcome	-	-
**Multicenter**	+	-	-	-	-	-	-	-
**Stratification**	Center and surgical technique	-	-	-	-	-	-	-
**Pulmonary risk stratification**	-	-	-	+	-	+	-	-
**Intervention**								
Duration of preoperative IMT	2-6 weeks	2 weeks	4 weeks	> 2 weeks	5 days	> 2 weeks	2-4 weeks	2 weeks
Supervised training sessions	1	1/week	1/week	1/week	5 sessions	1/week	1/week	1/week
High intensity program (≥ 60% MIP)	+	-	-	-	-	-	-	-
**Outcome**								
PPCs	+	+	-	+	-	+	-	-
Respiratory muscle function (pre-/postoperative)	+/+	+/+	+/−	+/−	+/+	+/−	+/+	+/+
Lung function (pre-/postoperative)	+/+	−/−	+/−	−/−	+/+	−/−	+/+	+/+

The PREPARE trial is the first study investigating the effect of preoperative IMT in a multicenter design. Despite increasing numbers of people with esophageal cancer, the number of patients undergoing esophageal resection surgery per surgical center are relatively low. To guarantee enough statistical power without the necessity of including patients for several years, a multicenter design was inevitable. The benefit of a multicenter design is that it optimizes external validity of the study allowing better generalization of the results. Furthermore, when IMT proves to be successful, information and practical experience of the intervention is already spread in several hospitals making implementation of IMT in routine care easier. The disadvantage of a multicenter trial is the handling with differences in daily care procedures. Every participating center has its own surgical and physical therapy protocol making the usual care in every center unique. However, by stratifying and randomizing per center, these differences in usual care will be evenly divided between both groups. Therefore the benefits of a multicenter approach weigh up to the disadvantages.

Besides the question whether a preoperative IMT program results in lower incidence of postoperative pneumonia, it is also relevant to investigate the causal mechanism of how IMT results in less pneumonia. When IMT is applied in the preoperative phase it may prevent pulmonary complications by decreasing the postoperative imbalance between increased ventilatory demand and decreased ventilatory capacity. The PREPARE protocol therefore included postoperative follow-up measurements of respiratory muscle and lung function, like the study of Dettling et al. [15]. The measurements will show whether the effects of preoperative IMT are still present in the postoperative phase and provide data for exploratory analyses on the possible working mechanism of how IMT may prevent PPCs.

The PREPARE study is the only study reporting a priori sample size calculation, and is unique in esophageal resection candidates with sufficient statistical power to show reductions in pneumonia events. All esophageal resection candidates are included in the PREPARE trial without considering individual risk for PPCs since at this point little is known about which patients will benefit the most from preoperative IMT. This is in contrast with two earlier studies who selected candidates for preoperative IMT based on a risk score for PPCs [13,16].

IMT is a simple intervention that is well tolerated by patients [33]. The researchers have chosen a home-based IMT program with guidance from a distance using weekly telephone consultations. This deviation of the other protocols where one training session a week is supervised (see Table 2) was chosen to decrease the burden for the patients and to increase inclusion rates. Patients often have to travel 30 minutes or more to reach the hospital. The standpoint of the PREPARE trial is that the intervention should fit in with daily care practice and with daily life of the patient. A weekly in-hospital physical therapy session of no more than 30 minutes (one high intensity training sessions of 30 breaths will take no more than 5 minutes) will discourage patients to participate in the trial or in an IMT program in general. Based on years of experience in guiding patients in performing their home-based IMT program, the PREPARE team presumes that clear instructions at baseline, an instruction video and the weekly telephone consultations should be sufficient to guide the patients and to monitor patient compliance. Furthermore, the memory of the digital inspiratory muscle trainer (POWERbreathe K3-series) enables the PTs to compare the last 36 trainings sessions with the recorded data in the patient diary which enables a supplementary check on patient compliance and adequate adjustment of the training load.

The PREPARE study is the first to use a high intensity training protocol in the preoperative phase. Based on promising results in healthy adults [34] and patients with COPD [14] the PREPARE team chose to use a high intensity training program proposed by Bailey et al. [18]. The protocol of 30 breaths twice daily is easy to perform unsupervised and will take only a few minutes per training session, possibly resulting in higher compliance to this specific training. The broad range of 5 to 200 cm H2O of the inspiratory load of the POWERbreathe equipment makes it possible to set the load for each individual. Other advantages of the POWERbreathe equipment is the tapered flow resistive loading, the gradually increasing load during the first three inspirations, the recording of the average load, average power, average volume, and training index (training effectiveness).

In conclusion, this multicenter RCT will investigate the hypothesis that preoperative IMT is associated with reduced postoperative pneumonia following esophageal resection. The PREPARE trial differentiates from other studies in its design and robustness.

### Trial status

Recruiting since September 2013.

## Abbreviations

ICU: Intensive care unit; IMT: Inspiratory muscle training; MIE: Minimally invasive esophageal resection; PPCs: Postoperative pulmonary complications; PT: Physical therapist; RCT: Randomized controlled trial; RPE: Rate of perceived exertion; THE: Transhiatal esophageal resection; TTE: Transthoracic esophageal resection; UPSS: Utrecht Pneumonia scoring system.

## Competing interests

The authors declare that they have no competing interests.

## Authors’ contributions

KV, JT, RG, RvH, and FB developed the study design. MS, JW, CR, HP, JS, SdH, JVR, EG, JR, ER, PN, MF, EK, MK, DLvdP, and SH made substantial contributions to the design of the trial. KV and JT drafted the manuscript. All authors provided input into revisions of the manuscript and have read and approved the final manuscript.
